# Lnc‐NA inhibits proliferation and metastasis in endometrioid endometrial carcinoma through regulation of NR4A1

**DOI:** 10.1111/jcmm.14345

**Published:** 2019-05-03

**Authors:** Linying Sun, Rongfang Zhou, Jing Dong, Shuang Liu, Yulian Jiao, Laicheng Wang, Shengnan Hu, Pengjuan He, Xiaowen Liu, Xingbo Zhao, Guosheng Jiang, Yueran Zhao

**Affiliations:** ^1^ Department of Central Lab Shandong Provincial Hospital affiliated with Shandong University Jinan China; ^2^ Institute of Public Health Taishan Medical University Taian China; ^3^ Department of Gynaecology and Obstetrics Shandong Provincial Hospital affiliated with Shandong University Jinan China; ^4^ Department of Immunology Binzhou Medical University Yantai China

**Keywords:** endometrial carcinoma, Lnc‐NA, migration and invasion, NR4A1, proliferation and apoptosis

## Abstract

Endometrioid endometrial carcinoma (EEC) is the most common gynaecologic malignancy worldwide. Long non‐coding RNAs have previously been demonstrated to play important roles in regulating human diseases, particularly cancer. However, the biological functions and molecular mechanisms of long non‐coding RNAs in EEC have not been extensively studied. Here, we describe the discovery of Lnc‐NA from the promoter of the transcription factor nuclear receptor subfamily 4 group A member 1 (NR4A1) gene. The role and function of Lnc‐NA in EEC remain unknown. In this study, we used quantitative real‐time polymerase chain reactions to confirm that Lnc‐NA expression was down‐regulated in 30 EEC cases (90%) and in EEC cell lines compared with that in the paired adjacent tissues and normal endometrial cells. In vitro experiments further demonstrated that overexpressing Lnc‐NA decreased EEC cell proliferation, migration and invasion and promoted apoptosis via inactivation of the apoptosis signalling pathway. Moreover, the results show that Lnc‐NA expression was positively correlated with NR4A1. Furthermore, Lnc‐NA regulated NR4A1 expression and activated the apoptosis signalling pathway to inhibit tumour progression. In summary, our results demonstrate that the Lnc‐NA‐NR4A1 axis could be a useful tumour suppressor and a promising therapeutic target for EEC.

## 1 INTRODUCTION

Long non‐coding RNAs (lncRNAs) are a class of non‐coding RNAs that are more than 200 nucleotides in length.^1^ Consistent with the wide range of lncRNA functions, several lines of evidence have demonstrated that lncRNAs participate in many human diseases,^2^, ^3^, ^4^ including cancer.^5^, ^6^, ^7^, ^8^, ^9^ Aberrant lncRNA expression may predict tumour outcomes in patients and serve as a diagnostic or prognostic marker. For example, lncRNA GClnc1 promotes gastric carcinogenesis by regulating gastric cancer cell growth and metastasis.^10^ LIMT is a novel metastasis‐inhibiting lncRNA that serves as a prognostic biomarker of breast cancer.^11^ MALAT1 is a lncRNA that mediates the tumour suppression functions of PCDH10 in endometrioid endometrial carcinoma (EEC) cells and is regulated by Wnt/β‐catenin signalling.^12^

The morbidity of endometrial cancer continues to increase worldwide, and endometrial cancer is the most common gynaecologic malignancy, particularly in China; however, an increasing number of advanced treatment strategies are being developed.^13^, ^14^ Endometrial cancers are divided into type I (hormone‐dependent) and type II (hormone‐independent). Type I EEC accounts for 80%–90% of all cases. Several studies have shown dysregulated expression and function of lncRNAs in EECs. For example, UCA1 has been reported as an oncogenic lncRNA in endometrial cancer, and silencing UCA1 inhibited endometrial cancer cell migration.^15^ lncRNA FER1L4 is down‐regulated in endometrial cancer and suppresses cancer cell proliferation by regulating PTEN expression.^16^ lncRNA HAND2‐AS1 inhibits invasion and metastasis in EECs through inactivation of neuromedin U.^17^ Nevertheless, the functions and clinical significance of lncRNAs in EEC remain unexplored. Therefore, we aimed to explore key lncRNAs that are involved in EEC progression.

In this study, we discovered the down‐regulation of a lncRNA, Lnc‐NA, in EEC. Lnc‐NA is a lncRNA‐transcribed antisense adjacent to nuclear receptor subfamily 4 group A member 1 (NR4A1) on chromosome 12q13.13. Several studies have reported NR4A1 as a tumour suppressor. For example, NR4A1 and Nor1 double knockout mice develop acute myeloid leukaemia.^18^ Some lymphomas have decreased NR4A1 expression, and NR4A1 overexpression in certain types of lymphoma cells induces apoptosis and inhibits the growth of these cell‐derived tumours in mice.^19^ NR4A1 also slows the growth of LNCaP prostate cancer cells.^20^ Ectopic NR4A1 expression in ERα‐expressing ZR‐75‐1 breast cancer cells and in PMC42 breast cancer cells with progenitor characteristics can inhibit cell migration, although proliferation and apoptosis in these cells are unaffected.^21^ Both Lnc‐NA and NR4A1 expression levels are down‐regulated in EECs. Our study thus reveals the functional role of Lnc‐NA in EEC.

## 2 METHODS

### 2.1 Clinical EEC specimens

This study was carried out according to the guidelines approved by the review board of the ethics committee of the Hospital of Shandong Provincial, Shandong University. Written informed consent was obtained from all the subjects in our research (In Supporting information). Pairs of EEC samples and adjacent normal endometrial tissues were collected from 30 patients who underwent a hysterectomy for EEC at the Department of Provincial Hospital affiliated with Shandong University. All adenocarcinoma samples were diagnosed and evaluated according to the International Federation of Gynecology Oncology criteria. The diagnoses of these EEC samples were verified by pathologists. Detailed pathologic and clinical data were collected for all samples.

### 2.2 Cell culture and transfection

The following cell lines were obtained from the China Type Culture Collection (Shanghai, China) and the Be Na Culture Collection (Beijing, China): KLE, RL95‐2, Ishikawa, HEC‐1‐b and ESC. Cells were grown in RPMI 1640 medium (Life Technologies, Inc, Grand Island, NY) supplemented with 10% FBS (Life Technologies, Inc) at 37°C in a humidified atmosphere of 5% CO_2_. Cell transfections were performed using Lipofectamine 3000 (Invitrogen, CA) according to the manufacturer's instructions.

### 2.3 Vector construction

The Lnc‐NA gene expression plasmid, the gene expression inhibitor plasmid and the respective empty vector plasmids were purchased from Shanghai GeneChem Co., Ltd. (Shanghai, China). Neomycin was used as the stable cell line selection marker.

### 2.4 RNA isolation and quantitative real‐time PCR

Total RNA was extracted using TRIzol reagent (Invitrogen, CA) according to the manufacturer's protocol. RNA quantity was measured with a NanoDrop ND‐2000 spectrophotometer (OD 260 nm, NanoDrop, Wilmington, DE), and RNA integrity was assessed using standard denaturing agarose gel electrophoresis. Lnc‐NA expression in tissue samples and cultured cells was quantified using SYBR Green I Master (Roche, Germany) and a LightCycler 480 (Roche Applied Science, Switzerland) according to the manufacturer's instructions. Fold changes were calculated using relative quantification (2^−∆∆Ct^), and human GAPDH was used as an internal control. The sequences of the primers used in the study are shown in Table S1. Each experiment was performed in triplicate.

### 2.5 Western blotting

Cells were lysed on ice with RIPA Lysis and Extraction Buffer (Thermo Fisher Scientific, USA) supplemented with protease and phosphatase inhibitors (Roche, Basel, Switzerland). Total protein samples were separated by electrophoresis with 10% SDS‐polyacrylamide gels and transferred onto PVDF membranes (Millipore, Billerica, MA). The membranes were blocked in 5% skim milk and incubated with primary antibodies (Table S2) overnight at 4°C, followed by incubation with secondary antibodies for 1 hour at room temperature. Then, the membranes were developed using an enhanced chemiluminescence (ECL) detection kit (Amersham Pharmacia Biotech, Piscataway, NJ). The relative amounts of various proteins were determined in relation to those of GAPDH.

### 2.6 Cell proliferation and apoptosis assays

2‐(2‐Methoxy‐4‐nitrophenyl)‐3‐(4‐nitrophenyl)‐5‐(2,4‐disulfothenyl)‐2H‐tetrazolium salt (CCK‐8, Dojindo, Rockville, USA) assays were conducted according to the manufacturer's instructions to evaluate cell proliferation rates. Briefly, log‐phase cells were trypsinized into a single‐cell suspension and plated into 96‐well plates at a density of 2 × 10^3^ cells per well. CCK‐8 solution was added to each well. After 1 h, the absorbance of each well at 450 nm was determined with a microplate reader (Enspire 2300 Multilabel Reader, Perkin Elmer, Singapore). For cell apoptosis analysis, cells were washed with PBS and resuspended in Annexin V binding buffer. Then, 5 μL of PE Annexin V and 5 μL of 7‐amino‐actinomycin (7‐AAD) (BD Biosciences, San Jose) were added to each sample and incubated for 15 minutes at room temperature in the dark according to the manufacturer's protocol. Immediately afterward, cell apoptosis was analysed with FACSCalibur flow cytometry (Becton Dickinson, Franklin Lakes, NJ).

### 2.7 Migration and invasion assays

For the cell migration assays, migration chambers (8‐μm pores) (Corning, Corning, NY) placed into a 24‐well plate were used. In the lower chamber, medium with 10% FBS was added as a chemoattractant. A total of 2 × 10^5^ cells were seeded on the upper insert in serum‐free medium. After incubation for 24 hours, the insert was fixed, stained, counted, and analysed using an inverted light microscope (Nikon). Each experiment was performed in triplicate. For the cell invasion assay, the procedure was similar to that of the cell migration assay, except the transwell membranes were coated with Matrigel (Corning).

### 2.8 Luciferase reporter assay

The NR4A1 promoter was PCR‐amplified from human genomic DNA and cloned into the pEZX‐LvPG04 Luciferase Reporter Vector (Genecopoeia). 293T cells were transfected with NR4A1 promoter plasmids or the control plasmid and Lnc‐NA plasmid. Forty‐eight hours following transfection, luciferase activity was analysed using the Secrete‐Pair^TM^ Dual Luminescence Assay Kits (Genecopoeia^TM^). The results were obtained from three independent experiments performed in duplicate.

### 2.9 Statistical analysis

Data are presented as the means ± SD. All statistical analyses were performed using ANOVA or a two‐tailed Student's *t *test to compare data. *P* < 0.05 was considered statistically.

## 3 RESULTS

### 3.1 Lnc‐NA was down‐regulated in human EEC tissues and cell lines and was bound to NR4A1

**Figure 1:**
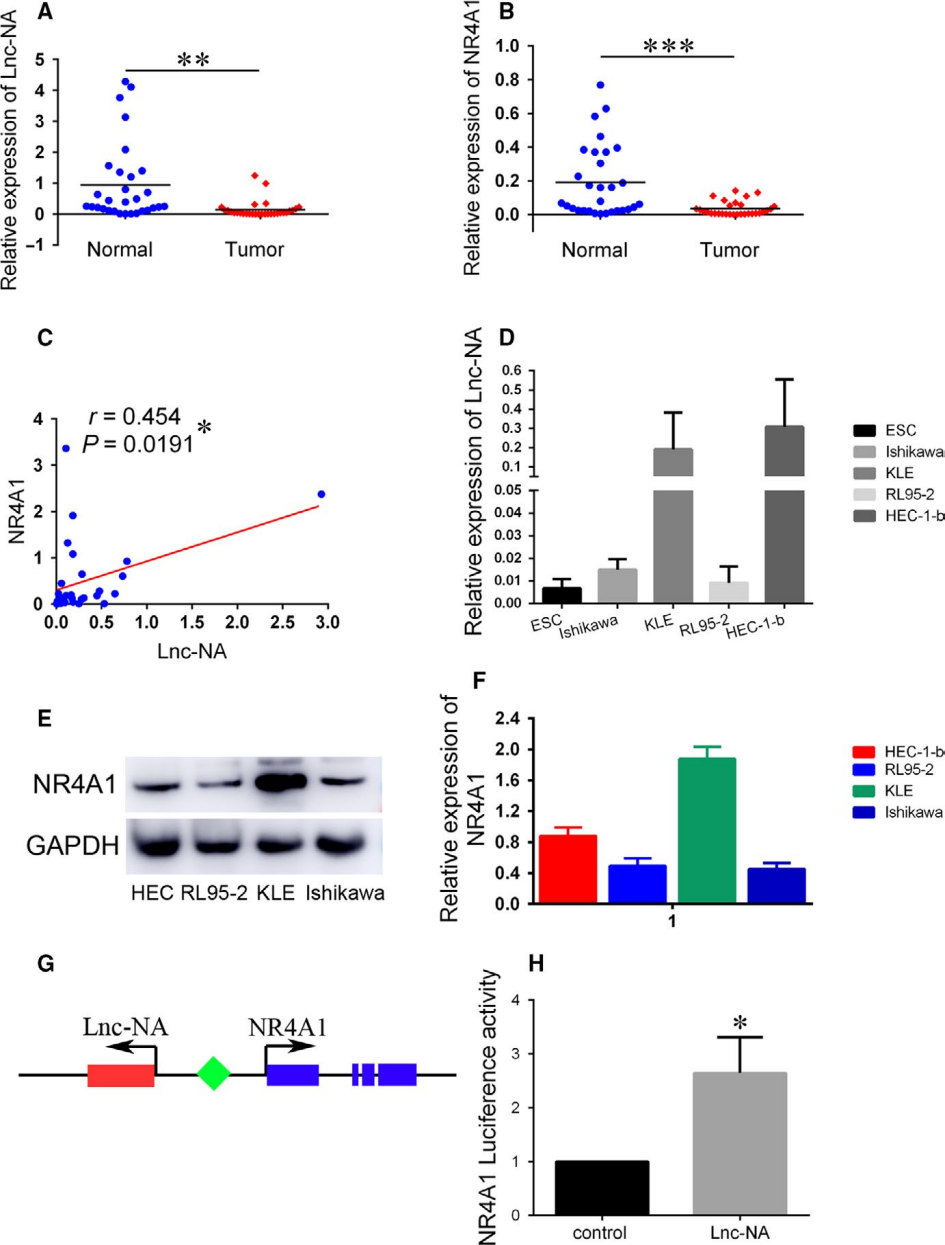
Relative Lnc‐NA and nuclear receptor subfamily 4 group A member 1 (NR4A1) expression in Endometrioid endometrial carcinoma (EEC) tissues and EEC cell lines. (A) Lnc‐NA expression levels in normal adjacent endometrial tissues (n = 30) and EEC tissues (n = 30) were examined by qRT‐PCR and normalized to GAPDH expression. (B) NR4A1 expression levels in normal adjacent endometrial tissues (n = 30) and EEC tissues (n = 30) were examined by qRT‐PCR and normalized to GAPDH expression. (C) Lnc‐NA and NR4A1 mRNA levels were positively associated in 30 EEC tissues (*r* = 0.454, *P* = 0.0191). (D) Lnc‐NA expression levels in EEC cell lines (n = 4) and normal endometrial cells were detected by qRT‐PCR. (E) NR4A1 expression levels in EEC cell lines (n = 4) were detected by Western blot. (F) Grey value analysis of the Western blot results. (G) Lasergene software was used to predict the region of NR4A1 that is anti‐sense to Lnc‐NA. (H) Luciferase activity assays in 293T cells showed the enhancement of NR4A1 promoter activity by Lnc‐NA (*P < 0.05). All data are shown as the means ± SD. Significant differences between groups are indicated as *P < 0.05; **P < 0.01; and *** *P* < 0.001

The functional role of Lnc‐NA in EEC is unknown. Therefore, we examined Lnc‐NA expression levels in EECs and characterized their clinical significance in EEC progression. We measured Lnc‐NA expression in 30 pairs of fresh EEC tissues and their adjacent matched normal tissues. We found that Lnc‐NA expression levels were significantly lower in the 30 EEC specimens than in the normal endometrial tissues (*P* = 0.0014, Figure 1A). To elucidate the relationship between Lnc‐NA and NR4A1, we used qRT‐PCR to measure the NR4A1 expression levels in our samples. NR4A1 was also down‐regulated in the EEC samples (*P* = 0.0002, Figure 1B). Furthermore, there was a significant correlation between Lnc‐NA and NR4A1 expression (*r* = 0.454, *P* = 0.0191) (Figure 1C). Additionally, in the EEC cell lines, Lnc‐NA expression levels were markedly higher in the KLE and HEC‐1‐b cells and lower in the Ishikawa and RL95‐2 cells (Figure 1D). The results for NR4A1 were similar; it was expressed at significantly lower levels in Ishikawa and RL95‐2 cells and at higher levels in KLE and HEC‐1‐b cells (Figure 1E,F).

To verify the binding sites of Lnc‐NA and NR4A1, we first analysed the reliability of Lnc‐NA through software and showed that the sequence of Lnc‐NA had a Fickett score of 0.367 34 with an incomplete putative open reading frame of 68 AA and a pI of 11.789 978 027 3, which, in total, classifies it as a non‐coding sequence with a coding probability of 0.179 828 9.^22^ Then, we used Lasergene software to predict the region of NR4A1 that is anti‐sense to Lnc‐NA. The combined area is approximately 383 bp (Table S3) and is located in the untranslated region of NR4A1 (Figure 1G). Subsequently, we confirmed their binding using luciferase reporter gene technology (Figure 1H). These results indicated that Lnc‐NA is positively correlated with NR4A1 expression in EECs.

### 3.2 Lnc‐NA inhibited cell proliferation and promoted cell apoptosis in EEC cells

**Figure 2:**
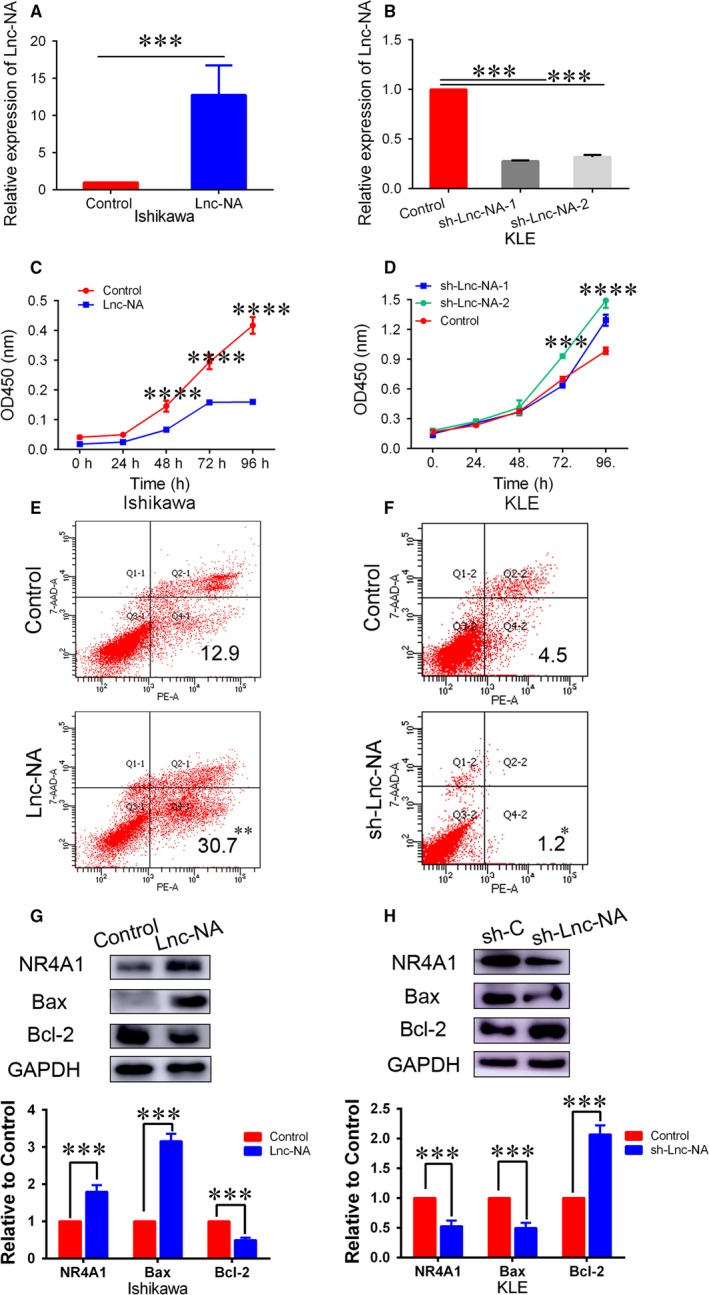
Lnc‐NA inhibited cell proliferation and promoted cell apoptosis in Endometrioid endometrial carcinoma cells. (A) qRT‐PCR analysis of Lnc‐NA expression levels after Lnc‐NA overexpression in Ishikawa cells. (B) qRT‐PCR analysis of Lnc‐NA expression levels after Lnc‐NA knockdown in KLE cells. (C) CCK‐8 assays were performed to determine cell proliferation activities after transfection for 0, 24, 48, 72, and 96 h. The data show that compared with the negative groups, Lnc‐NA overexpression significantly decreased cell proliferation. (D) CCK‐8 assays were performed to determine cell proliferation activities after transfection for 0, 24, 48, 72, and 96 h. The data show that, compared with the negative groups, Lnc‐NA knockdown significantly increased cell proliferation. (E) FACS analysis was used to determine the per cent of Annexin V+/7‐AAD‐ (apoptotic) cells in Ishikawa cells transfected with the Lnc‐NA construct or negative control. (F) FACS analysis was used to determine the per cent of Annexin V+/7‐AAD‐ (apoptotic) cells in KLE cells transfected with the sh‐Lnc‐NA construct or negative control. (G) nuclear receptor subfamily 4 group A member 1 (NR4A1), Bcl2 and Bax expression levels in Ishikawa cells after transfection with the Lnc‐NA construct or negative control were determined by Western blot. (H) NR4A1, Bcl2, and Bax expression levels in Ishikawa cells after transfection with the sh‐Lnc‐NA construct or negative control were determined by Western blot. All data are shown as the means ± SD, n = 3. Significant differences between groups are indicated as *P < 0.05, **P < 0.01, ***P < 0.001, and ****P < 0.0001

To elucidate the underlying mechanisms of Lnc‐NA in EEC development, we performed in vitro assays to examine the effects of Lnc‐NA on cell proliferation and apoptosis in EEC cells. Thus, we selected and transfected two recipient EEC cell lines: Ishikawa cells were transfected with Lnc‐NA or Lnc‐NA‐control, and KLE cells were transfected with Lnc‐NA‐inhibitor or Lnc‐NA‐inhibitor‐control. Cell lines with Lnc‐NA overexpression and knockdown were established and designated Ishikawa‐Lnc‐NA and KLE‐sh‐Lnc‐NA. As shown in Figure 2A, Lnc‐NA expression levels were significantly higher in the Lnc‐NA plasmid‐transfected group than those in the control group (*P* < 0.001). However, compared with control siRNA transfection, sh‐Lnc‐NA transfection reduced Lnc‐NA expression levels (*P* < 0.001).

**Figure 3:**
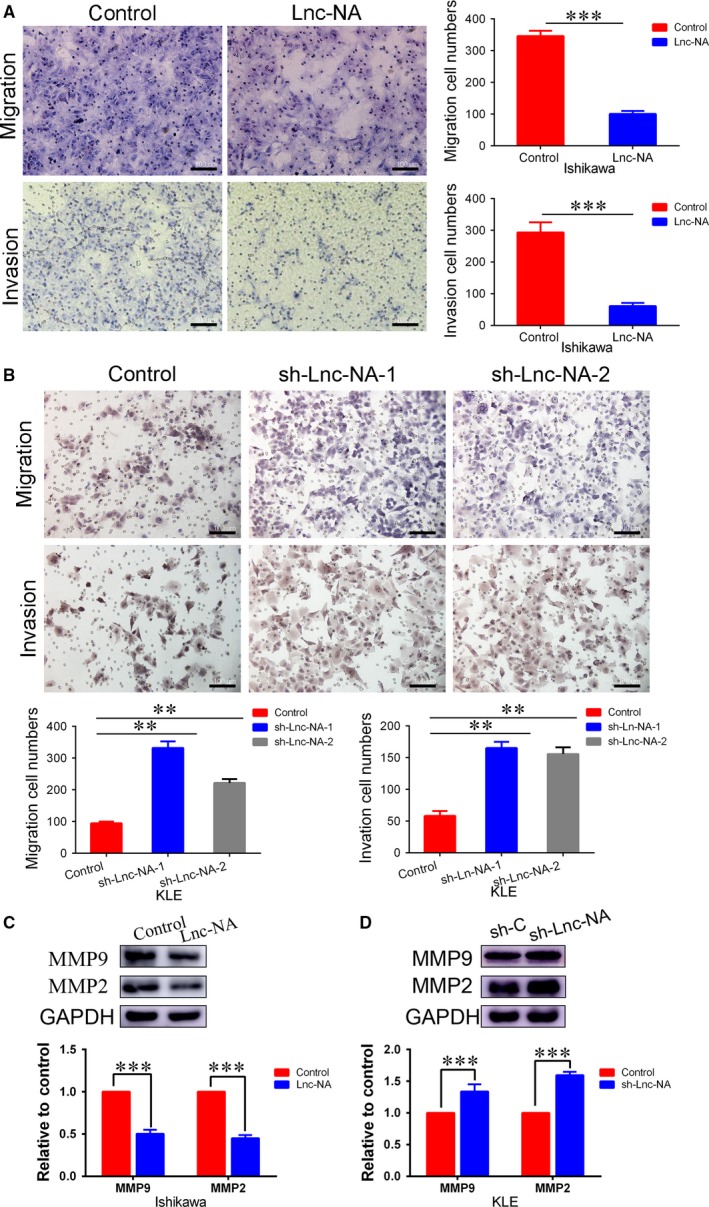
Lnc‐NA inhibited cell invasion and migration in Endometrioid endometrial carcinoma cells. (A) Transwell assays were performed to assess migration and invasion capabilities. The data indicate that compared with the controls, Lnc‐NA overexpression markedly suppressed migration and invasion. (B) Transwell assays were performed to assess migration and invasion capabilities. The data indicate that compared with the controls, Lnc‐NA knockdown markedly promoted migration and invasion. (C) Western blotting indicated that MMP2 and MMP9 protein expression was down‐regulated in the Lnc‐NA group compared with that in the control group. (D) Western blotting indicated that MMP2 and MMP9 protein expression was up‐regulated in the sh‐Lnc‐NA group compared with that in the control group. All data are shown as the means ± SD, n = 3. Significant differences between groups are indicated as **P < 0.01, and ***P < 0.001

The effects of Lnc‐NA on EEC cell growth were determined by CCK‐8 assays. Ishikawa cells transfected with Lnc‐NA had lower growth index values (OD values at 450 nm) at 48, 72, and 96 hours post‐transfection than those transfected with Lnc‐NA‐control (Figure 2C; *P* < 0.001). In contrast, KLE cells transfected with sh‐Lnc‐NA had higher growth index values at 72 and 96 hours post‐transfection than those transfected with sh‐Lnc‐NA‐control (Figure 2D; *P* < 0.001). The effects of Lnc‐NA on cell apoptosis in EEC cells were determined by flow cytometry. As shown in Figure 3E, compared with the control groups, Lnc‐NA overexpression in Ishikawa cells promoted cell apoptosis (*P* < 0.01), whereas Lnc‐NA knockdown in KLE cells inhibited cell apoptosis (*P* < 0.05).

To further analyse the relationship between Lnc‐NA and NR4A1, we examined NR4A1 expression after Lnc‐NA up‐regulation and down‐regulation. Our results showed that compared with the controls, Lnc‐NA overexpression increased NR4A1 expression levels, and Lnc‐NA knockdown decreased NR4A1 expression levels (Figure 2G,H, *P* < 0.001). Studies have shown that Bcl2/Bax are involved in apoptosis in tumours.^23^, ^24^, ^25^ Since our study showed that Lnc‐NA promotes apoptosis in EEC cells, we evaluated the relationship between Lnc‐NA and Bcl2/Bax. As shown in Figure 2G, Lnc‐NA overexpression increased Bax expression levels and decreased Bcl2 expression levels (*P* < 0.001). However, Lnc‐NA knockdown inhibited Bax expression and promoted Bcl2 expression. Our results suggest that Lnc‐NA inhibits cell progression and promotes cell apoptosis and that Lnc‐NA and NR4A1 expression levels are consistent in EEC cells.

### 3.3 Lnc‐NA inhibited cell invasion and migration in EEC cells

After verifying the inhibitory role of Lnc‐NA in EEC cell viability, we further investigated whether this lncRNA participates in cell migration and invasion. Compared with Ishikawa cells transfected with the negative vector, cells transfected with Lnc‐NA for 48 hours showed significantly decreased migration ability (Figure 3A, *P* < 0.001). Ishikawa cells also exhibited significant impairments in invasion ability after transfection with Lnc‐NA for 48 hours (Figure 3A, *P* < 0.001). In addition, we performed migration and invasion assays to examine the effects of Lnc‐NA knockdown on cell migration and invasion ability in KLE cells. Compared to the controls, Lnc‐NA knockdown caused a significant increase in the number of migrated and invaded cells (Figure 3B, *P* < 0.01).

Matrix metalloproteinases play an important role in tumour invasion and migration.^26^, ^27^ Therefore, we determined the effects of Lnc‐NA on MMP2 and MMP9. Western blotting showed that MMP2/9 protein expression levels were down‐regulated in the Lnc‐NA group (Figure 3C, *P* < 0.001). In contrast, MMP2/9 expression was up‐regulated in Lnc‐NA knockdown KLE cells compared with that in control cells.

Collectively, these data suggest that Lnc‐NA decreased cell invasion and migration in EEC cells.

### 3.4 NR4A1 is a target of Lnc‐NA

**Figure 4:**
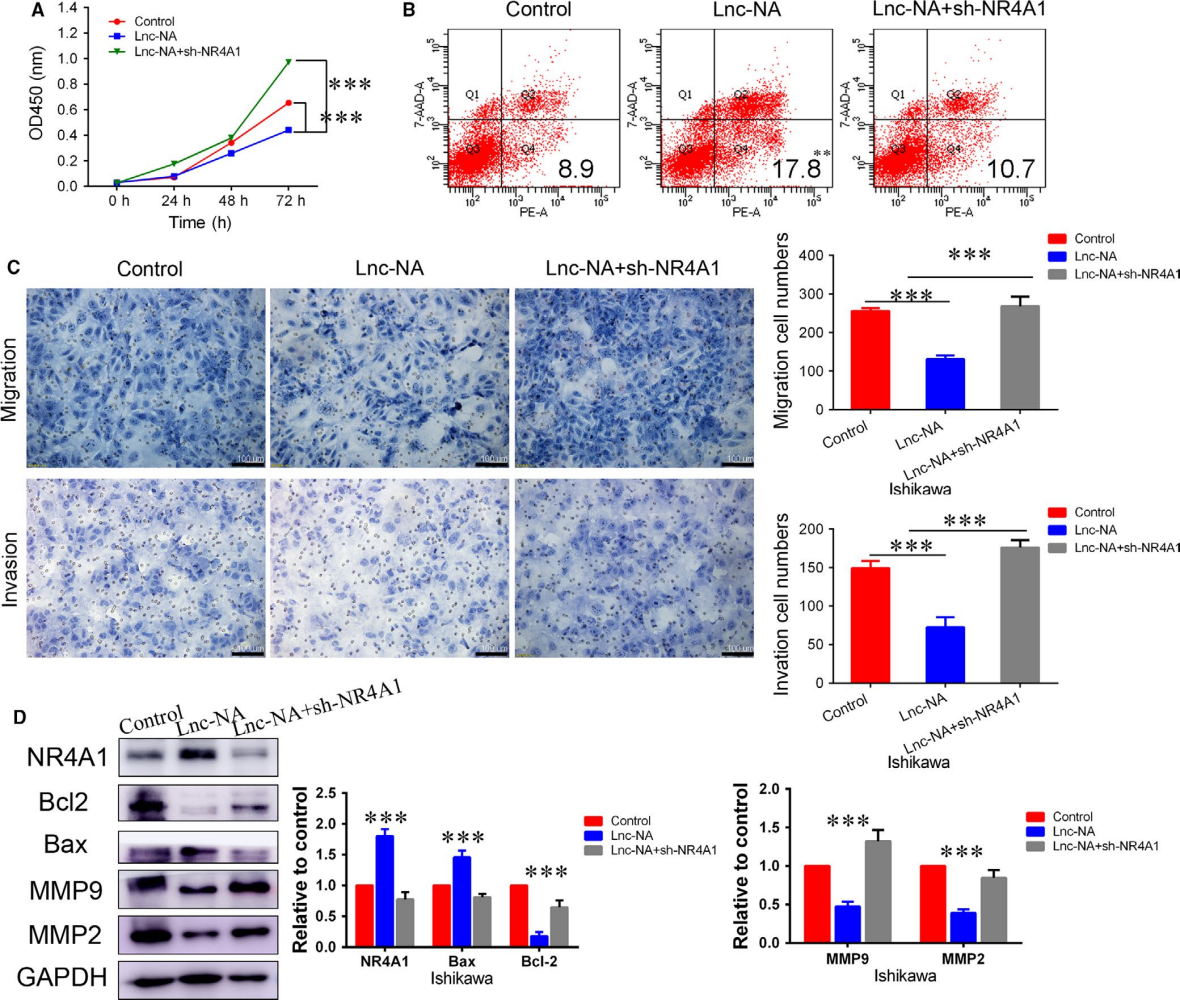
Nuclear receptor subfamily 4 group A member 1 (NR4A1) is a target of Lnc‐NA. (A) The effects of NR4A1 on cell proliferation after NR4A1 knockdown in Ishikawa‐Lnc‐NA cells were evaluated using the CCK‐8 assay. (B) The effects of NR4A1 on cell apoptosis after NR4A1 knockdown in Ishikawa‐Lnc‐NA cells were evaluated using FACS. (C) The effects of NR4A1 on migration and invasion in Ishikawa‐Lnc‐NA cells were determined using migration and invasion assays. (D) Western blots show NR4A1, Bax, Bcl2, MMP2/9 protein expression levels when NR4A1 was knocked down in Ishikawa‐Lnc‐NA cells. All data are shown as the means ± SD, n = 3. Significant differences between groups are indicated as **P < 0.01, and ***P < 0.001

To determine the importance of NR4A1 in Lnc‐NA‐mediated proliferation, apoptosis, migration, and invasion in EEC cells, we silenced NR4A1 expression in the Ishikawa‐Lnc‐NA cell line. First, CCK‐8 assay results showed that compared with the Ishikawa‐Lnc‐NA group, NR4A1 knockdown significantly increased cell proliferation (Figure 4A, *P* < 0.001). Next, we performed flow cytometry analyses to determine the role of NR4A1 in cell apoptosis. As shown in Figure 4B, the inhibitory effects of Lnc‐NA on apoptosis in EEC cells were significantly decreased after NR4A1 knockdown (*P* < 0.01). In addition, the migration and invasion assay results showed that cell numbers were higher in NR4A1 knockdown cells than in Ishikawa‐Lnc‐NA cells (Figure 4C, *P* < 0.001). As a control, we examined the role of NR4A1 overexpression in Ishikawa cells, and the results showed that overexpression of NR4A1 inhibited cell proliferation, migration, and invasion while promoting cell apoptosis (Figure S1, *P* < 0.05). Meanwhile, to demonstrate that Lnc‐NA exerts its effects by targeting NR4A1, we measured NR4A1, Bcl2/Bax, and MMP2/9 expression levels by Western blot. Consistent with the cell function experiment results, Bcl2 expression levels were increased, and Bax expression levels were decreased upon NR4A1 knockdown. NR4A1 knockdown increased MMP2 and MMP9 protein expression levels compared with those of the control group (Figure 4D, *P* < 0.001).

**Figure 5:**
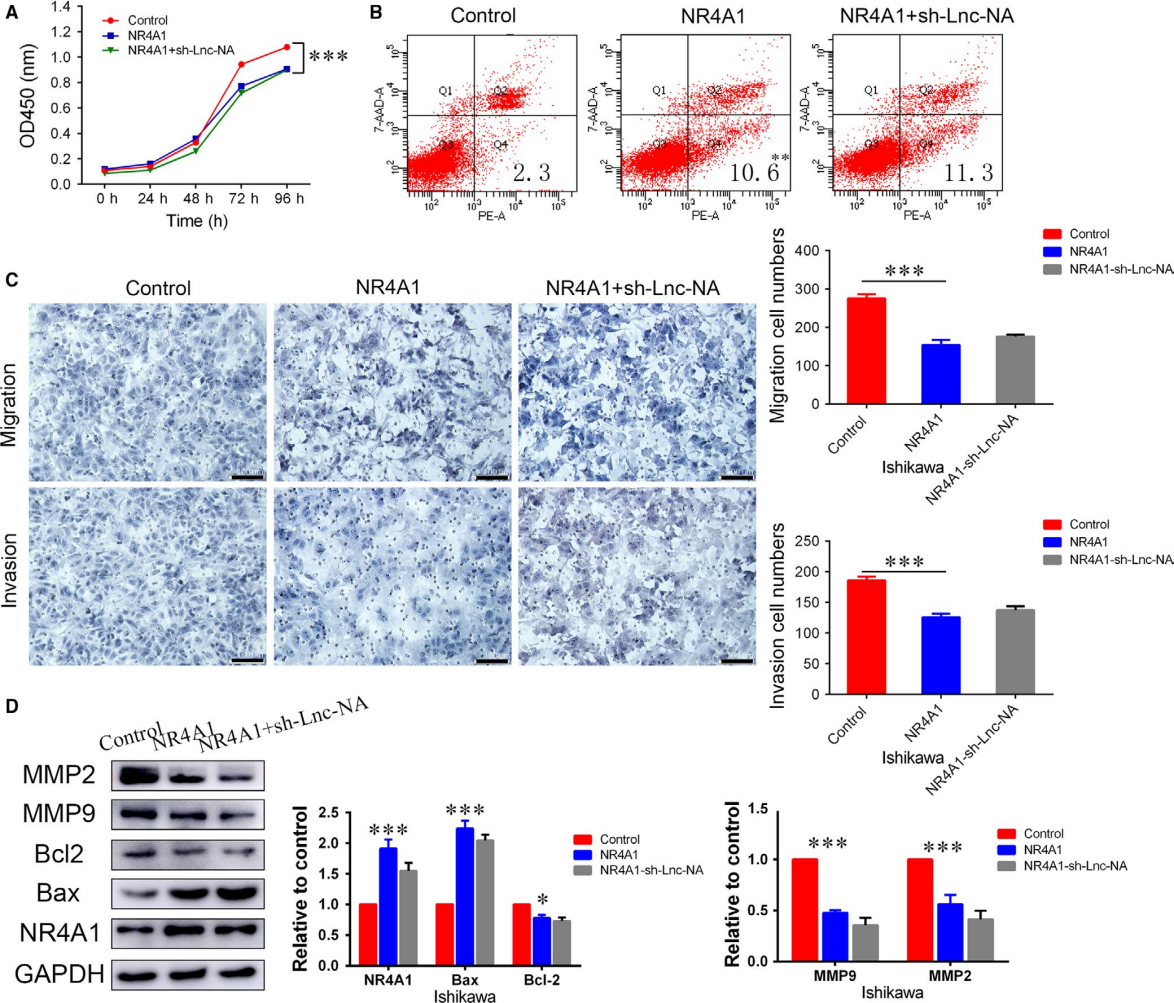
Knockdown of Lnc‐NA did not reverse the phenotype of nuclear receptor subfamily 4 group A member 1 (NR4A1)‐overexpressing cells. (A) CCK‐8 assays were performed to determine cell proliferation activities after transfection for 0, 24, 48, 72 h and 96 h. The data show that compared with the negative groups, knockdown of Lnc‐NA did not increase cell proliferation. (B) The effects of sh‐Lnc‐NA on cell apoptosis in NR4A1‐overexpressing Ishikawa cells were evaluated using FACS. (C) The effects of sh‐Lnc‐NA on migration and invasion in NR4A1‐overexpressing Ishikawa cells were determined using migration and invasion assays. (D) Western blots show NR4A1, Bax, Bcl2, and MMP2/9 protein expression levels when Lnc‐NA was knocked down in NR4A1 overexpressing Ishikawa cells. All data are shown as the means ± SD, n = 3. Significant differences between groups are indicated as *P < 0.05, **P < 0.01, and ***P < 0.001

Furthermore, we examined the effect of down‐regulating Lnc‐NA in cell lines overexpressing NR4A1. We found that Lnc‐NA did not reverse the proliferation, apoptosis, invasion, and migration of cells caused by overexpression of NR4A1. At the same time, the results of Western blotting indicate that down‐regulation of Lnc‐NA did not alter the associated protein expression (Figure 5, *P* < 0.05). These data suggested that NR4A1 is a target of Lnc‐NA, which inhibits the progression of EEC by promoting the expression of NR4A1.

### 3.5 The Lnc‐NA‐NR4A1 axis promotes apoptosis signalling

**Figure 6:**
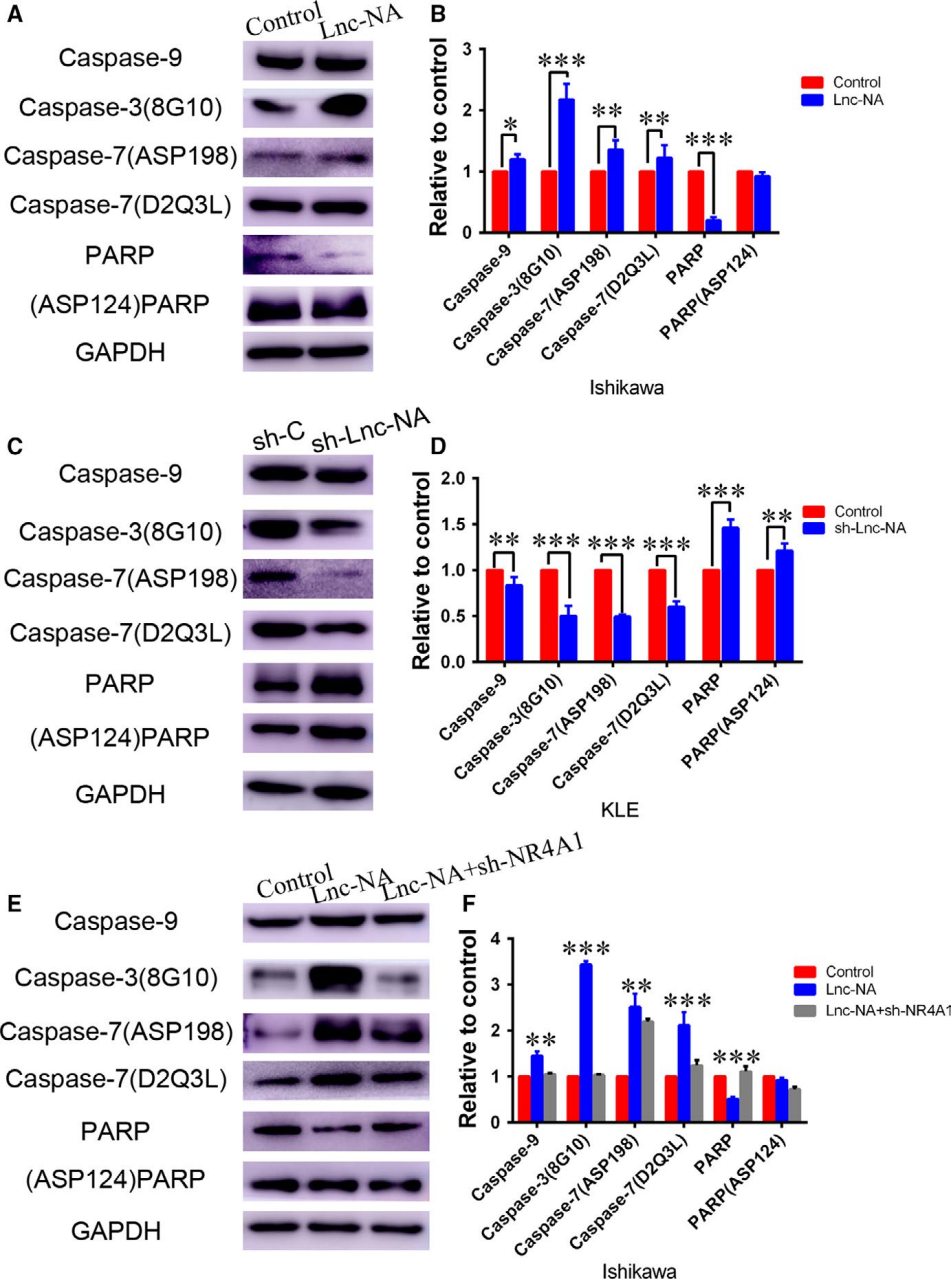
The Lnc‐NA‐NR4A1 axis functions by promoting apoptosis signalling. (A) Western blots show caspase 9, cleaved‐caspase3 (8G10), caspase 7 (D2Q3L), cleaved‐caspase 7 (ASP198), PARP and cleaved‐PARP (ASP124) protein expression levels when Lnc‐NA was up‐regulated in Ishikawa cells. (B) Densitometry analysis of the Western blotting results is shown in (A). (C) Western blots show caspase 9, cleaved‐caspase3 (8G10), caspase 7 (D2Q3L), cleaved‐caspase7 (ASP198), PARP and cleaved‐PARP (ASP124) protein expression when Lnc‐NA was down‐regulated in KLE cells. (D) Densitometry analysis of the Western blotting results shown in (C). (E) Western blots show caspase 9, cleaved‐caspase 3 (8G10), caspase 7 (D2Q3L), cleaved‐caspase7 (ASP198), PARP and cleaved‐PARP (ASP124) protein expression levels when NR4A1 was knocked down in Ishikawa‐RP3.8 cells. (F) Densitometry analysis of the Western blotting results is shown in (E). All data are shown as the means ± SD, n = 3. Significant differences between groups are indicated as *P < 0.05, **P < 0.01, and ***P < 0.001

**Figure 7:**
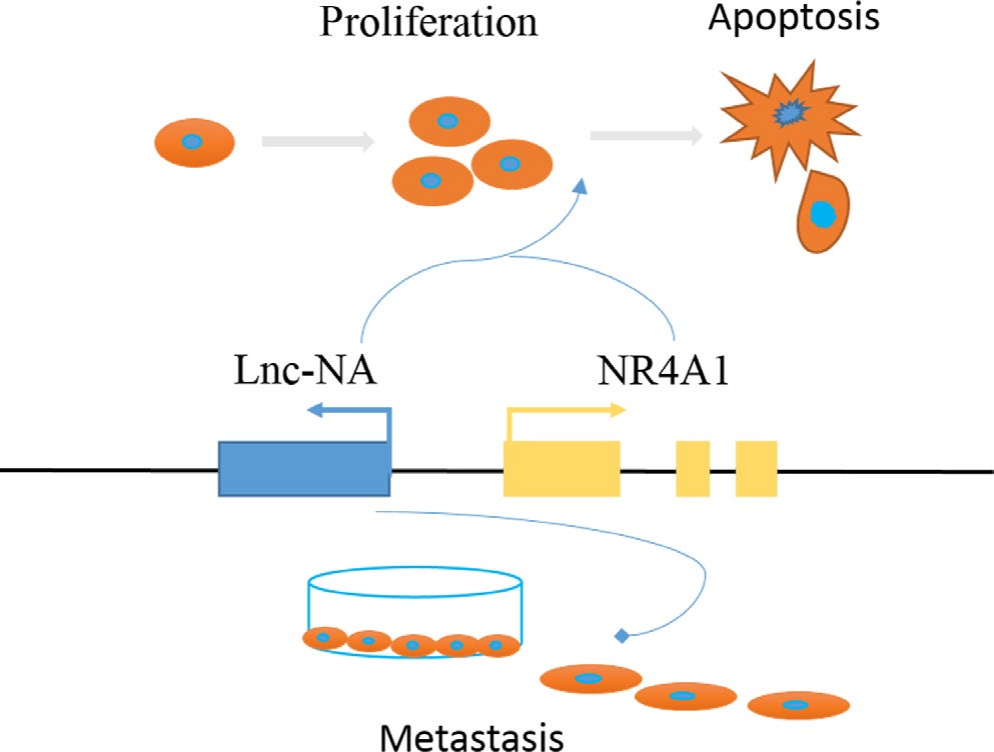
A model of Lnc‐NA in Endometrioid endometrial carcinoma (EEC). The model depicts the role of Lnc‐NA in EEC. When differentiation starts, Lnc‐NA (blue) is concurrently transcribed upstream of the nuclear receptor subfamily 4 group A member 1 (NR4A1) gene (yellow). Lnc‐NA binds to NR4A1, promoting the transcription and translation of NR4A1, which then leads to the activation of target genes. Lnc‐NA also inhibited the invasion and migration of EEC cells by regulating NR4A1‐independent function. Other aspects of Lnc‐NA regulation remain unclear and will be explored in the future

Accumulating evidence indicates that NR4A1 decreases tumour cell growth by triggering apoptosis signalling in many solid tumours.^28^, ^29^, ^30^ To investigate the underlying mechanism by which the Lnc‐NA‐NR4A1 axis regulates cell growth, apoptosis, migration, and invasion, we determined the expression of proteins involved in apoptosis signalling. Our results showed that when Lnc‐NA was up‐regulated, the protein expression levels of caspases 9, 3, and 7 increased, whereas PARP protein expression levels decreased (Figure 5A,B). Furthermore, in the Lnc‐NA knockdown KLE cell line, the protein expression levels of caspases 9, 3, and 7 decreased, whereas PARP protein expression levels increased (Figure 6C,D). To demonstrate that Lnc‐NA exerts its effects by targeting NR4A1, we measured the expression levels of caspases 9, 3, and 7 and PARP by Western blot. The results showed that down‐regulation of NR4A1 can reverse the promotion of apoptosis of Lnc‐NA (Figure 6E,F). Therefore, the results indicate that the Lnc‐NA‐NR4A1 (Figure 7) axis inhibited proliferation in EEC cells by activating the apoptotic signalling pathway.

## 4 DISCUSSION

An accumulating amount of evidence indicates that multiple lncRNAs play important roles in regulating gene expression in normal cells. The dysregulation of lncRNAs may be involved in epigenetics and may participate in many key cellular processes in tumour cells, such as cell growth, differentiation, invasion, metastasis, and death.^31^ Mounting evidence has emphasized the roles and clinical significance of lncRNAs in cancer. Several known lncRNAs (HOTAIR, GAS5, and UCA1) have recently been reported to be differentially expressed in EEC tissues and involved in EEC progression and metastasis.^32^, ^33^ In this study, the analysis of 30 EEC samples revealed that lncRNA Lnc‐NA is significantly down‐regulated in EEC tissues compared with normal or benign tissues. The role of Lnc‐NA was studied in EEC for the first time. In vitro mechanistic studies demonstrated that Lnc‐NA inhibited cell proliferation, invasion, and migration. In addition, Lnc‐NA also promoted cell apoptosis. These findings suggest that Lnc‐NA may be useful as a prognostic marker for patients with EEC.

NR4A1 (Nur77, TR3), NR4A2 (Nurr1), and NR4A3 (NOR‐1) are nuclear orphan receptors of the Nur77 family and are widely expressed in different types of tissues, such as skeletal muscle, adipose tissue, heart, kidney, T cells, liver, and brain. As recently described, these receptors are also involved in tumorigenesis.^34^, ^35^ Therefore, we analysed NR4A1 expression in EEC tissues and EEC cell lines. The results showed that low levels of NR4A1 were also present in patients and cell lines with low levels of Lnc‐NA. These findings indicate that Lnc‐NA and NR4A1 expression are correlated in EEC patients. An analysis of the data indicated that Lnc‐NA and NR4A1 are located on the same chromosome and that Lnc‐NA is adjacent to NR4A1. Furthermore, we verified the relationship between Lnc‐NA and NR4A1 in cell lines. The results show that Lnc‐NA can up‐regulate NR4A1 expression. In addition, we found that NR4A1 overexpression inhibits cell proliferation, invasion, and migration and promotes cell apoptosis. Therefore, our study shows that Lnc‐NA can inhibit the progression of tumours by possibly affecting NR4A1 expression.

Some studies suggest that upstream antisense RNAs may exert some biological functions by regulating corresponding genes.^36^, ^37^ To further verify that NR4A1 expression plays a role in Lnc‐NA‐mediated cellular biological functions, we down‐regulated NR4A1 in Lnc‐NA‐overexpressing cells. The results of CCK‐8 assays showed that NR4A1 knockdown can reverse the inhibitory effects of Lnc‐NA on cell proliferation. The apoptosis analysis results indicated that down‐regulating NR4A1 inhibited the Lnc‐NA‐mediated promotion of tumour cell apoptosis. In addition, our migration and invasion results were consistent with the CCK‐8 assay results. Furthermore, we measured Bcl2/Bax and MMP2/9 expression after NR4A1 knockdown. On the other hand, to demonstrate that Lnc‐NA is upstream of NR4A1 in the Lnc‐NA‐NR4A1 axis, we down‐regulated the expression of Lnc‐NA in NR4A1‐overexpressing cells. The results show that the knockdown of Lnc‐NA could not reverse the phenotype of NR4A1‐overexpressing cells. Thus, our results demonstrate that Lnc‐NA inhibits cell proliferation, migration, and invasion and promotes cell apoptosis by targeting NR4A1.

Although NR4A1 target genes are poorly characterized, the proapoptotic effects of NR4A1 may be achieved by its transactivation function of acting as a transcription factor (TF) for apoptosis‐inducing genes.^38^, ^39^, ^40^, ^41^ Consequently, proteins in the extrinsic and intrinsic apoptosis pathways, including TRAIL, Puma, and all isoforms of Bim, have already been identified as potential targets of NR4A1 in an NR4A1 transgenic mouse model.^42^, ^43^ These results indicate that NR4A1 plays an important role in tumour apoptotic signalling. Therefore, we examined the role of the Lnc‐NA‐NR4A1 axis in EEC apoptosis. The results showed that the expression levels of caspase 9/3/7 and PARP, key proteins in the apoptotic signalling pathway, were changed correspondingly after Lnc‐NA up‐regulation, that is, Lnc‐NA‐NR4A1 signalling activated the apoptotic signalling pathway in EEC. Furthermore, we demonstrated that Lnc‐NA activates the expression of apoptotic signalling pathway‐related proteins by altering NR4A1 expression, which in turn promotes cell apoptosis.

Several genome‐wide studies have suggested that promoters of protein‐coding genes are the origins of LncRNA transcription.^44^ Our studies indicate that Lnc‐NA binds to the upstream portion of the promoter of the TF NR4A1. This is in line with a recent report showing that divergent transcription is associated with promoters of transcriptional regulators.^36^ This seems to be common, at least during myogenic differentiation, but there are still few studies in other areas. Our report provides evidence that supports this phenomenon. However, how the lncRNA moves within the nucleus and specifically binds with the TF to guide it to or remove it from the transtargets remains to be determined. Mechanistic research needs to continue in the future.

In conclusion, we discovered that lncRNA Lnc‐NA is frequently down‐regulated in EECs. Lnc‐NA overexpression decreased proliferation, migration, and invasion and increased apoptosis in EEC cells by up‐regulating NR4A1. Furthermore, our findings indicate that the Lnc‐NA‐NR4A1 axis plays a tumour suppressing role in EEC progression by activating apoptosis signalling. Therefore, the Lnc‐NA‐NR4A1 axis could serve as a candidate prognostic marker for EEC.

## CONFLICT OF INTEREST

The authors declare no conflicts of interest for this article.

## AUTHORS’ CONTRIBUTIONS

LY Sun and YR Zhao designed the experiments; LY Sun carried out all of the experiments and data collection; RF Zhou, S Liu, LC Wang, PJ He, and XW Liu helped with the collection of clinical samples. LY Sun wrote the manuscript and prepared the figures; J Dong, SN Hu, YL Jiao, and XB Zhao provided technical support; LY Sun, YR Zhao, and GS Jiang revised the manuscript. All authors approve and agree to be responsible for all aspects of this work.
