# Supplementary figures and images for: Lnc‐NA inhibits proliferation and metastasis in endometrioid endometrial carcinoma through regulation of NR4A1
Source: J Cell Mol Med. 2019 May 3;23(7):4699–710. doi: 10.1111/jcmm.14345 (PMC6584524; doi:10.1111/jcmm.14345)

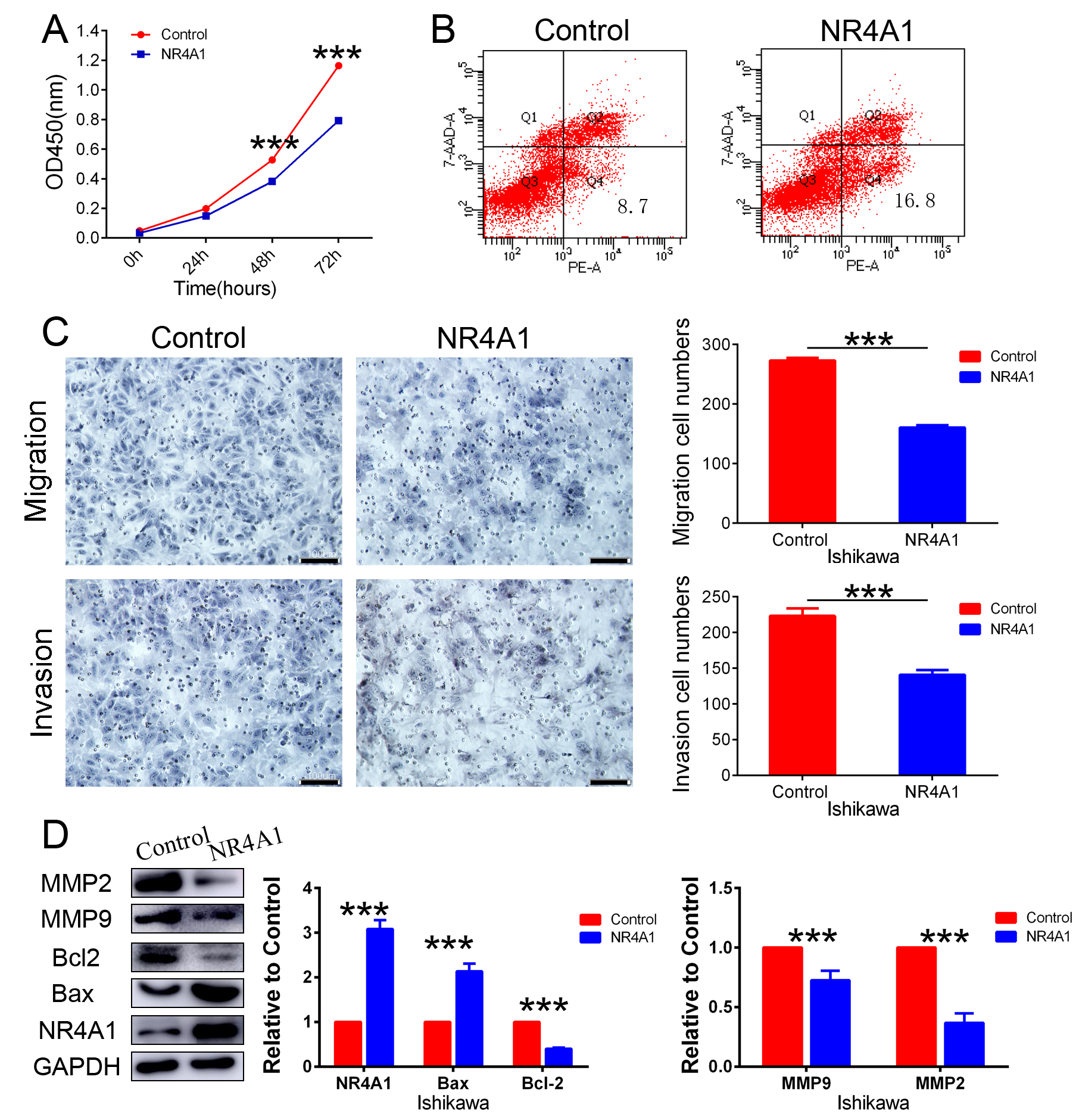

Supplement: Supplementary file 1 [file JCMM-23-4699-s001.tif]

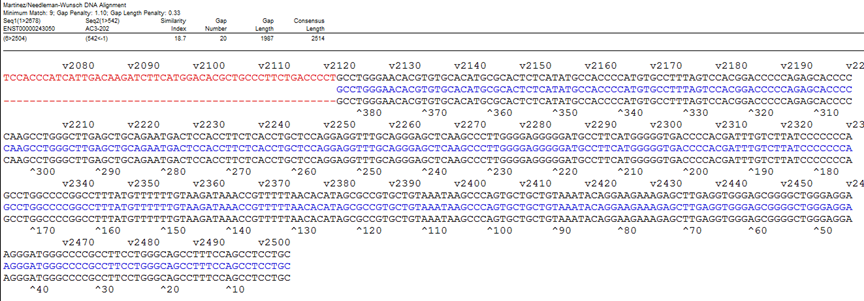
Table S3 Lasergene softwar predicts the binding position of Lnc-NA and NR4A1.

Supplement: Supplementary file 4 [file JCMM-23-4699-s004.doc]
