# Supplementary material for: Lnc‐NA inhibits proliferation and metastasis in endometrioid endometrial carcinoma through regulation of NR4A1
Source: J Cell Mol Med. 2019 May 3;23(7):4699–710. doi: 10.1111/jcmm.14345 (PMC6584524; doi:10.1111/jcmm.14345)
Supplement: Supplementary file 2 [file JCMM-23-4699-s002.doc]

# Table S1. The sequences of primer used for qRT-PCR

| Gene | Forward primer(5’-3’) | Reverse primer(5’-3’) |
| --- | --- | --- |
| Lnc-NA | GCATTCACATGTTGGCCATTAG | TCGGGTTCTCACTTGGAGTAT |
| NR4A1 | AGCATTATGGTGTCCGCACAT | TTGGCGTTTTTCTGCACTGT |
| GAPDH | ACAGTCAGCCGCATCTTCT | GACAAGCTTCCCGTTCTCAG |
