# Supplementary material for: Lnc‐NA inhibits proliferation and metastasis in endometrioid endometrial carcinoma through regulation of NR4A1
Source: J Cell Mol Med. 2019 May 3;23(7):4699–710. doi: 10.1111/jcmm.14345 (PMC6584524; doi:10.1111/jcmm.14345)
Supplement: Supplementary file 3 [file JCMM-23-4699-s003.doc]

**Table 2S. Antibodies used in this study**

| **Antibody** | **Source** | **Concentration** | **Product Number** | **Manufacture** |
| --- | --- | --- | --- | --- |
| NR4A1 | Rabbit | 1:1000 | Ab-52039 | Abcam |
| Bcl-2 | Rabbit | 1:1000 | Sc-15393 | Santa Cruz |
| Bax | Rabbit | 1:1000 | ab33168 | Abcam |
| MMP2 | Rabbit | 1:1000 | ab92536 | Abcam |
| MMP9 | Rabbit | 1:1000 | ab76003 | Abcam |
| Caspase9 | Mouse | 1:1000 | #9915 | Cell signaling Technology |
| Cleaved-Caspase3 (8G10) | Rabbit | 1:1000 | #9915 | Cell signaling Technology |
| Caspase 7  (D2Q3L) | Rabbit | 1:1000 | #9915 | Cell signaling Technology |
| Cleaved-Caspase7 (ASP198) | Rabbit | 1:1000 | #9915 | Cell signaling Technology |
| PARP | Rabbit | 1:1000 | #9915 | Cell signaling Technology |
| Cleaved-PARP (ASP124) | Rabbit | 1:1000 | #9915 | Cell signaling Technology |
| GAPDH | Rabbit | 1:2000 | Sc-25778 | Santa Cruz |

Note: WB: Western blot.
